# Variability of the mitochondrial *CO1* gene in native and invasive populations of *Harmonia axyridis* Pall. comparative analysis

**DOI:** 10.1371/journal.pone.0231009

**Published:** 2020-04-02

**Authors:** Alla Blekhman, Irina Goryacheva, Dimitry Schepetov, Ilia Zakharov

**Affiliations:** 1 Russian Academy of Sciences, Koltzov Institute of Developmental Biology, Moscow, Russia; 2 Russian Academy of Sciences, Vavilov Institute of General Genetics, Moscow, Russia; 3 Moscow Regional State University, Moscow, Russia; University of São Paulo, BRAZIL

## Abstract

Our study is focused on original and publicly accessible data on the intraspecific variability of the barcoding DNA fragment in ladybirds *Harmonia axyridis* Pall analysis. The complete dataset consists of 39 haplotypes, 16 of which we identified for the first time. The intra-population and geographical variability of the barcoding fragment was studied for seven populations of the western and eastern groups of the native range and in six invasive populations, in which 25 of the 39 haplotypes are found. Population structure inferred on base of molecular variability and haplotype frequencies showed a high level of differences between the eastern and western groups of native populations and confirm the hypothesis of the origin of all invasive populations from native populations of the eastern group. A comparative analysis of molecular variation indices testifies to various evolutionary scenarios of the formation of the western and eastern groups of native populations and confirms the hypothesis of the microevolutionary history of the species, previously suggested in morphological character based studies of the geographical variability of *H*. *axyridis*. A significant decrease in the molecular diversity of invasive populations confirms the hypothesis of a random nature of the primary invasion of this species in North America.

## Introduction

*Harmonia axyridis* (Pallas, 1773) (Coleoptera, Coccinellidae) is a wide-areal species that before the 90s. of the last century inhabited Central and Northeast Asia. The boundaries and features of the native range of this species based on the cadastral list of finds and their localization on the map were described in detail [1–3]. A significant part of the native range of the species is located in Russia–from the middle course of the Irtysh River in Western Siberia to the Pacific coast, including Sakhalin, and the South Kuril Islands. In the north, the species range reaches 57° – 58° north. lat., apparently following the boundaries of the permafrost zone. In the south-west, the border of the range captures the forest-steppe zone of north-eastern Kazakhstan, then passes along the upper reaches of the Irtysh River in the north-western part of Mongolia, and turns south, enveloping the desert and semi-desert regions of Mongolia and north-west of China, as well as the Tibetan plateau, reaching the province of Szechwan. Further southward propagation of the species is putatively limited by the tropical climate zone. In northeast and central China, the Korean Peninsula, and the Japanese islands, *H*. *axyridis* is abundant.

*H*. *axyridis* has attracted the attention of evolutionary biologists and has become a classic object of population-genetic studies of microevolutionary processes since the beginning of the XX century, primarily due to the amazing variability of the pattern and coloring of their elytra. The study of phenogeography of this species was begun by F.G. Dobrzansky [4] and then continued by different researchers on different parts of the range in Japan [5–7], in China and Korea [7,8], as well as in Russia [9–11]. To date, an analysis of the geographical variability of *H*. *axyridis* by a complex of three morphological characters (elytral pattern, pronotum pattern, elytral ridge) [12–14] has shown that this species (within the native range) has an interesting intraspecific structure. Within the species range, there are two large groups of populations geographically distant from each other–Western and Eastern, the differences between which according to the 75% criterion [15] correspond to a subspecies level. Information on the differences in the phenotype of these population groups is given in the supplement (S1 Fig and S2 Fig). The western group includes populations living west of Lake Baikal, as well as in Altai, and in the Sayans. The eastern group includes populations living on a vast territory–east of East Transbaikalia in Russia, in China, Korea and Japan. In Central Siberia, in Western Transbaikalia, there is a zone of clinically variable morphological characters. Studies of *cytochrome oxidase gene subunit 1 (CO1)* 5P fragment (1490–2198 bp) polymorphism, in selected Russian populations of the western and eastern groups [13, 16] and analysis of 18 microsatellite loci in 9 native populations of Kazakhstan, Russia, China, Korea, and Japan [17] confirmed the existing differences between populations of the western and eastern groups. The formation of the modern intraspecific structure of *H*. *axyridis* is probably associated with the emergence of long-term isolation between eastern and western populations during the Sartan glaciation (25–10 thousand years ago) [13, 14, 18–20].

Apart from purely scientific interest, *H*. *axyridis* acquired significant economic importance in recent years. Due to ecological plasticity and wide polyphagy, this species, which is one of the most voracious predators of aphids and a number of other plant parasite insects [21], has become a very popular biocontrol agent. Numerous releases of *H*. *axyridis* beetles and larvae in order to combat agricultural pests have been undertaken in the USA since 1916 [22], in Russia and Eastern Europe (Carpathian region)–from the middle of the XX century [23, 24], in Western Europe–since the beginning of the 80s of the last century [25]. Despite the fact that numerous animals were released for many years throughout the past century, the first *H*. *axyridis* population that wintered in nature outside its native range was discovered only in 1988 in the USA, in New Orleans, Louisiana, 360 km from the last place in this state to release them for biocontrol in 1973 [26]. This fact made it possible to speculate about the random nature of the introduction of *H*. *axyridis* in Louisiana by sea transport and its origin from one of the natural populations of China [27]. Soon, by the end of the last century, this ladybird had already spread across both American continents [22, 28]. Since the beginning of our century, natural invasive populations of *H*. *axyridis* have appeared in countries of Western Europe, including those where there were no artificial release events [29], as well as in South Africa [30]. Thus, in just two decades, the invasion of this species, covering four continents, has become global [31, 32]. *H*. *axyridis* has evolved from a useful biocontrol agent into an aggressive invasive species that poses a serious threat to biodiversity of indigenous ecosystems, as well as certain sectors of the economy and human health [22, 33]. Currently, *H*. *axyridis*, whose invasive range continues to expand [34–39], is one of the most popular natural model objects (species) for studying various aspects of biological invasions.

Comparative studies of the polymorphism of individual morphological and molecular genetic markers in *H*. *axyridis* made it possible to establish some features of the genetic structure of invasive populations and their putative origin. In particular, data on allozyme polymorphism in *H*. *axyridis* populations from 8 different states of the USA (from 7 states of the Atlantic coast and from Oregon–the Pacific coast) [40] showed that all studied populations come from a single, but quite a large source. The assumption of the common origin of the North American populations of *H*. *axyridis* is supported by data on the geographical variability of the elitrae pattern, an important morphological character for this species. US populations are almost monomorphic in the pattern of elitraes with the *succinea* phenotype [22, 40]. Beetles of the *conspicua* phenotype were found in Denver with a frequency of less than 1% [41] [38]. In the southwestern state of Oregon, the proportion of this phenotype in nature was 1.4%; in the same state, the only specimen of the *spectabilis* phenotype was found from almost 3,000 beetles of the biocontrol line imported from Japan [42, 43]. The pheno-pattern of invasive populations of Europe turned out to be significantly more polymorphic. In European populations, the main mass of *H*. *axyridis* is also composed of the succinea phenotype beetles; however, unlike America, the proportion of melanists (*conspicua* and *spectabilis*) here is quite large and varies from 12% to 28% in different populations, which suggests their mixed origin [29]. In general, according to polymorphic morphological characters, invasive populations are very close to the eastern group of native range populations and differ sharply from the populations of the western group [41]. Published data on the variability of molecular genetic markers in *H*. *axyridis* populations, both native and invasive, is incomplete. Only one haplotype of the barcoding fragment (5ʹ-fragment of the *CO1* gene) was found in three geographically distant American populations of *H*. *axyridis*–from Oregon (Pacific coast) and Kentucky (Atlantic coast) [44]. A study of the polymorphism of the same molecular marker [13] in the populations of Gorno-Altaysk (the western group of the native range), Vladivostok (the eastern group of the native range) and Denver showed significant differences between the populations of Gorno-Altaysk and Vladivostok, a low level of population variability Denver and its high similarity and difference with the populations of Vladivostok and Denver, respectively. Comparative analysis of polymorphism of a shorter than the barcoding fragment of the 5'-region of the *CO1* gene (567 bp) in four invasive (Denver, Berlin, Turin, Cambridge) and three native (Gorno-Altaysk, Vladivostok, Kyoto) populations [16] also showed a high similarity between invasive populations and native populations of the eastern group.

Evaluation of different *H*. *axyridis* invasion scenarios based on the variability of the system of 18 microsatellite loci in native, invasive, and biocontrol populations showed that two bridgehead populations initially occurred as a result of two independent introductions in the east and west of the United States. The eastern bridgehead has become the likely source of four introductions–to Africa, South America (Brazil/Argentina and Chili) and Europe, and the western bridgehead–is the source of at least two independent introductions to Europe [17, 45, 46]. European invasive populations could also have been formed as a result of hybridization of ladybugs from the eastern North American bridgehead and the biocontrol line imported to Europe in 1982 from China, with a probability of the given scenario of 0.8134. The scenario of the origin of the North American invasion centers themselves, according to microsatellite analysis, was somewhat unexpected. The populations of the western center are based on natives of East Asian populations of the native range, which is in good agreement with the above data on the variability of morphological characters and the 5'-fragment of the *CO1* gene. The eastern center, which became the “waystation” for global invasion, turned out to be phylogenetically associated with both the eastern and western groups of the native range populations, which clearly contradicts the above results and conclusions of studies on mitochondrial and morphological markers. Most likely, mitochondrial and nuclear molecular genetic markers are necessary for the final decision on the origin of the “bridgehead” population of *H*. *axyridis* in eastern North America, because their polymorphism marks more ancient evolutionary events than too variable microsatellite loci.

Despite considerable efforts to determine the origin of invasive populations and biology features [47], which provided *H*. *axyridis* with rapid continental settlement, the molecular genetic studies of native *H*. *axyridis* populations, in which preadaptations to invasions realized at the end of the XX century have not been carried out to date.

The main scope of our work was a comparative study of the genetic structure of invasive and native *H*. *axyridis* populations from different geographical regions. Despite the fact that approaches based on new genotyping technologies are finding wider application in population genetics, we used a barcoding fragment (5'-end of the *CO1* gene), since data on its structure are most widely represented in international databases, and data on the structure of other molecular markers are scarce.

## Material and methods

### DNA sampling

The material for this work was the collection of *H*. *axyridis* from 6 natural populations of the invasive range and from 7 populations representing the western and eastern populations of the native range of the species [13, 14]. Caught beetles were frozen and stored at -20° C until DNA isolation. Data on collectors, locations and dates of collection, the number of extracted and analyzed DNA samples, as well as their storage locations are presented in Table 1.

**Table 1:** Material characteristics.

Population status		Collection site	Date of collection	Collector	Number of DNA samples
Invasive		Denver (USA)	September 2004	I.A. Zakharov-Gezekhus	25*
		Munchen (Germany)	October 2006	I.A. Zakharov-Gezekhus	21*
		Berlin (Germany)	July 2008	I.A. Zakharov-Gezekhus	21*
		Prague (Czech Republic)	November 2011	I.A. Zakharov-Gezekhus	23*
		Kaliningrad (Russia)	August 2010	I.A. Zakharov-Gezekhus	26*
		Sochi (Russia)	May 2013	M.Y. Orlova-Benkovskaya	26** (2)
Native	Western group	Novosibirsk (Russia)	September 2006	I.A. Zakharov-Gezekhus	36**
		Gorno-Altask (Russia)	September 2005	I.A. Zakharov-Gezekhus	31*
		Sayan Mountains (Russia)	October 2011	I.A. Zakharov-Gezekhus	33**
	Eastern group	Birobidzhan (Russia)	Autumn 2009	L.V Frisman	30**
		Vladivostok (Russia)	Autumn 2009	V.P. Korablev	34**
		The Russky Island (Russia)	October 2003	A.V. Zimenko	30**
		The Troitsa Bay (Russia)	October 2003	A.V. Zimenko	30**
		Total			366

*DNA samples are stored in Insect DNA Collection, Vavilov Institute of General Genetics

**DNA samples are stored in Collection of polymorphic species Coccinellidae bioresource collection of the Koltsov Institute of Development Biology.

### DNA isolation, amplification, electrophoresis, elution, sequencing

Total DNA was isolated from the abdomen of beetles using the DIAtom DNA Prep DNA isolation kit from Isogen (Russia) in accordance with the manufacturer's manual, but with a lysis time increased to 2 hours. The isolated DNA was eluted with NucleoS^TM^, a highly purified deionized sterile water sorbent for molecular genetic studies. The concentration of the obtained DNA samples was measured on a NanoDrop8000 microspectrophotometer (Termoscientific). Isolated DNA samples were stored at—20° C (Table 1).

Amplification of the 5ʹ-fragment of the cytochrome oxidase 1 (*Cox1*) gene (barcoding fragment) was carried out by PCR with a pair of primers that we developed specifically for *H*. *axyridis*: F3457 5'–GACATTGGAACATTATACCTTTTA–3' and R79133 5'–AATTTTTTTCCCTCTTTCTTGTGT–3'. In comparison to the traditionally used primers LCO1490 and HCO2198 [48] [45], our pair allows us to obtain a highly specific PCR product with a length of 759 bp, without unspecific byproduct and avoid cleaning samples on an agarose gel. The resulting product includes a traditional barcoding fragment with the exception of the first eleven nucleotides of the 5'-end of the sequence, which allows using the array of previously obtained data available in GenBank for comparative analysis. The amplification reaction was carried out in a final volume of 25 μl containing 30–50 ng of total DNA, 2mM MgCl2, 0.2mM of each dNTP, 4 pmol of each primer, 1 unit of HS Taq polymerase DNA (Eurogen, Russia) and PCR buffer in a single final concentration. PCR was performed on an ABI Veriti 96 well thermal cycler (Applied Biosystems, USA) under the following conditions: initial denaturation– 4 min at 95° C; then 5 cycles using the auto-delta function: denaturation– 30 sec at 94° C, annealing– 50 sec with a temperature decrease from 60° C to 56° C at 10° C in each cycle, starting from the second, polymerization– 50 sec at 72° C; then 34 cycles followed: denaturation– 50 sec at 94° C, annealing– 50 sec at 580C and polymerization– 50 sec at 72° C. Then followed the final polymerization– 5 min at 72° C.

PCR success was evaluated by electrophoresis of 3 μl of the reaction mixture in 1.5% agarose gel stained with ethidium bromide. To clean the PCR product, kits were used to isolate DNA from agarose gel and Cleanup Mini reaction mixtures manufactured by Eurogen (Russia) in accordance with the manufacturer's manual.

Sequencing of purified mtDNA amplicones in both directions was carried out at the IDB RAS CCU using the ABI 3500 Genetic Analyzer. For the sequencing reactions, the same primers were used as in the amplification reaction with Big Dye Terminator reagent kit (Applied Biosystems, v. 3.1). Processing of the obtained nucleotide sequences was carried out using the SeqMan software package Lasergene 11 (DNASTAR, USA). Ambiguously decrypted positions were validated manually. The resulting sequences were aligned using the MUSCLE algorithm and trimmed for further analysis to a length of 647 bp. in the MEGA X software package [49]. In addition to the sequences that we obtained in this study, we also used all *CO1 H*. *axyridis* sequences from the GenBank nucleotide database, which fully include the fragment we are analyzing and that do not have ambiguous or N letters and / or stop codons in translation (104 sequences in total). The GenBank sequences were used only for assessing the general parameters of the intraspecific polymorphism of the barcoding fragment (*CO1*) (nucleotide composition, the total number of haplotypes and their distribution over the species range, as well as for phylogenetic analysis of haplotypes).

### Phylogenetic analysis

To estimate the phylogenetic relationships of the mitotypes recovered, a Maximum Likelihood analysis was performed using the Tamura 3-parameter model, which was identified as the best replacement model based on Bayesian information criterion (BIC) in MEGA X [49]. The stability of ML dendrogram nodes was estimated by the bootstrap method with 1000 replicates.

Phylogenetic reconstructions were also performed using the posterior probability (BI) estimation method in MrBayes [50] v. 3.2.5 using the same predefined model of molecular evolution. Reconstructions were carried out in 12 million generations and completed with a standard deviation of the divided frequencies of 0.002.

As an external group, seven *CO1 Harmonia quadripunctata* sequences from the GenBank were used, with the following accession numbers (ID): KM446380, KM447142, KU906663, KU909968, KU914822, KU915124, KU917400.

To assess the intraspecific phylogenetic relationships of the detected haplotypes, the median network was built using the Network 5.0 program [51], the MJ algorithm (median-joining) using the Optional Postprocessing/MP (maximum parsimony) Calculation option.

### Population genetic analysis

AMOVA estimation of fixation indices (FST) and their P-value, haplotype (Hd) and nucleotide (π) diversity, as well as the average number of pairwise differences (Pi) were performed in the Arlequin v. 3.5.1.2 software package [52] with molecular distance estimation using the pairwise differences. Cluster analysis of samples on the distribution of haplotypes in populations was carried out in Statistica 6.0 software package (Stat Soft, Inc., USA) with the Single Linkage method using the Squared Euclidean distances square matrix.

## Results

### Amplification and sequencing

We obtained *CO1* gene fragments from 366 samples, with length of 672–740 bp, depending on read quality. All sequences translate without insertions, deletions, or stop codons. Aligned and trimmed to 647 bp sequences split into 25 haplotypes. One sequence of each haplotype is deposited into the nucleotide database of the GenBank with registration numbers (ID): MN509563 –MN509583. *CO1* sequences of *H*. *axyridis* available from the GenBank nucleotide database, include 14 more haplotypes.

Analysis of the molecular variability of all 39 identified haplotypes showed that this fragment is AT-rich, the average total nucleotide ratio is– 15.5%–C: 38.41%–T: 30.92%–A: 15.14%–G. The sequences of the identified haplotypes contain 35 polymorphic sites (5.4%), 36 substitutions, of which 24 are transitions and 12 transversions. In nine haplotypes at eight polymorphic sites, eight non-synonymous substitutions occur, leading to substitutions of amino acid residues in the protein sequence. Details of polymorphic sites and the nature of substitutions for each haplotype are shown in S1 Table.

### Phylogenetic analysis

**Fig 1:**
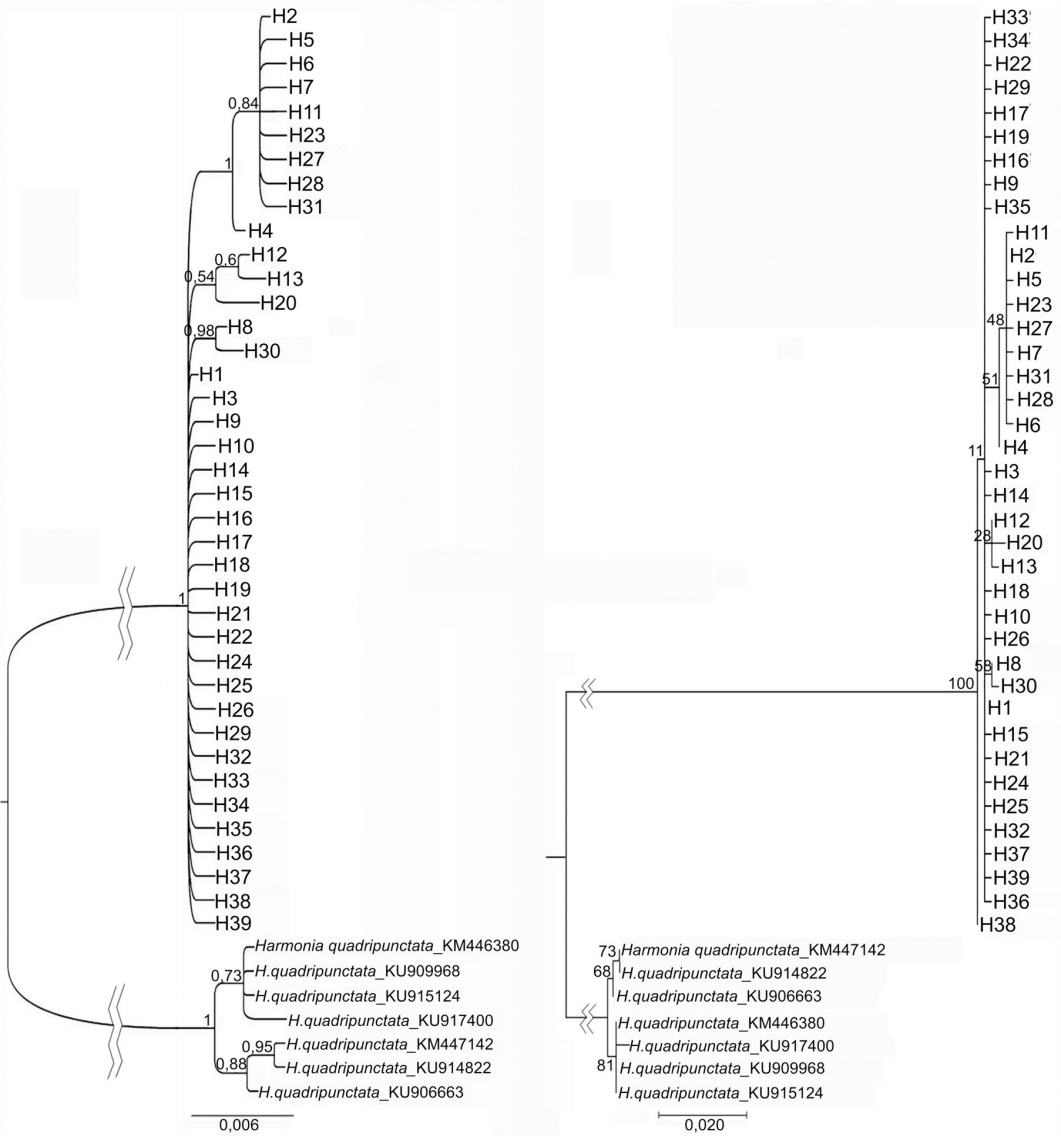
*CO1* haplotype cladogram for *H*. *axyridis*, based on: A–maximum likelihood method (ML) under Tamura 3-parameter model of molecular evolution; and B–Bayesian estimation of posterior probability (BI) under GTR+G+I model of molecular evolution.

The results of the analysis of the phylogenetic relationships of the identified haplotypes by the Maximum Likelihood (ML) method and the estimation of the posterior probabilities (BI) are presented in Fig 1. Despite the low support for some nodes, both dendrograms have a similar topology: a large cluster of haplotypes one substitution away from the H1 haplotype, and a smaller cluster including 10 haplotypes: H4 (closest to the main pool of haplotypes), H2 and equidistant from it H5, H6, H7, H11, H23, H27, H28, H31. In both dendrograms, a cluster of three haplotypes H12, H13 and H20 and a group of two close haplotypes H8 and H30 are also present.

**Fig 2:**
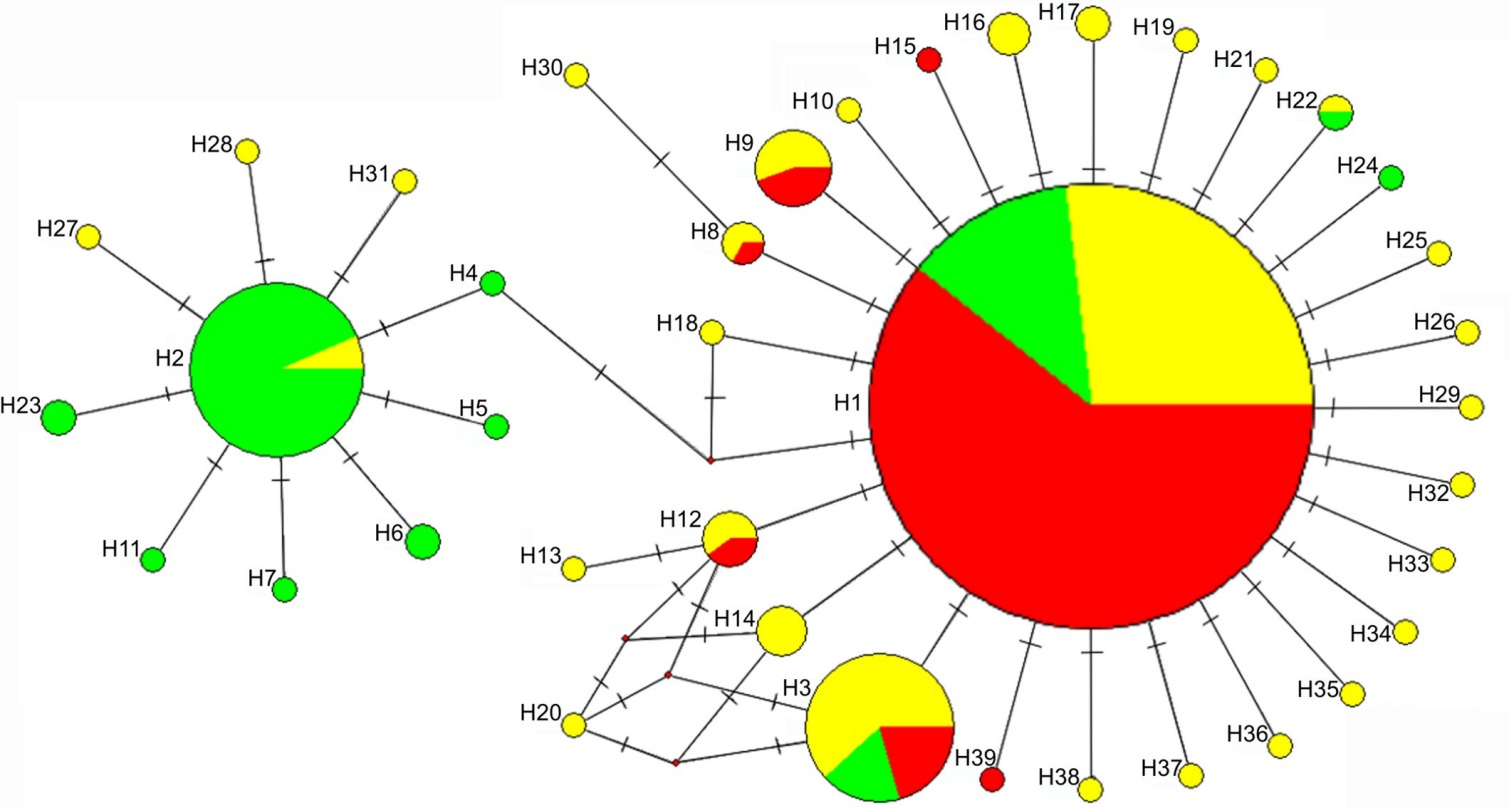
Haplotype network of the *CO1* gene from *H*. *axyridis* specimens. Circles represent haplotypes, circle size denotes the total haplotype frequency, while slices represent the haplotype frequencies in different population groups. Color red represents invasive populations, color green represents native western populations, color yellow represents native eastern populations.

The median haplotype network (Fig 2) shows a similar result: two star-shaped groups with H1 and H2 in the centers, interconnected to the H4 haplotype, characterized by two mutational steps from H1 and one replacement from H2, short chains of H1 –H8 haplotypes–H30 and H1 –H12 –H13, each having one substitution in each link and the H20 haplotype, distant from H1 by three mutational steps with alternative linkage possible.

### Intraspecific diversity and geographical distribution of haplotypes

The results of the analysis of the occurrence and intraspecific distribution of haplotypes obtained by comparative analysis of 470 *H*. *axyridis CO1* sequences (including 104 sequences from GenBank) are shown in Tables 2 (according to our data) and 3 (according to GenBank), as well as colouration on Fig 2.

**Table 2:** Distribution of haplotypes in populations / population groups of *H*. *axyridis*.

Population haplotype	Native western population group				Native western population group					Invasive							Total
	**Novosibirsl**	**Gorno-Altaysk**	**Sayans**	***Mitotype proportion in group in %***	**Birobidzhan**	**Vladivostok**	**The Russky Island**	**The Troitsa Bay**	***Mitotype proportion in group in %***	**Sochi**	**Prague**	**Berlin**	**Kaliningrad**	**Munich**	**Denver**	***Mitotype proportion in group in %***	
**H1**	12	11	18	**41**	22	28	19	16	**68.6**	24	18	19	25	21	23	**91.5**	**256**
**H2**	16	14	13	**43**													**43**
**H3**	5	1		**6**	4		10	6	**16.1**	1	3					**2.8**	**30**
**H4**		1		**1**													**1**
**H5**		1		**1**													**1**
**H6**		1	1	**2**													**2**
**H7**		1		**1**													**1**
**H8**						1			**0.8**						1	**0.7**	**2**
**H9**						3		1	**3.2**		1	1	1		1	**2.8**	**8**
**H10**						1			**0.8**								**1**
**H11**		1		**1**													**1**
**H12**							1	1	**1.6**	1						**0.7**	**3**
**H13**								1	**0.8**								**1**
**H14**								4	**3.2**								**4**
**H15**												1				**0.7**	**1**
**H16**					1				**0.8**								**1**
**H17**					1				**0.8**								**1**
**H18**					1				**0.8**								**1**
**H19**					1				**0.8**								**1**
**H20**								1	**0.8**								**1**
**H21**						1			**0.8**								**1**
**H22**	1			**1**													**1**
**H23**	2			**2**													**2**
**H24**			1	**1**													1
**H25**											1					**0.7**	1
Sample size	36	31	33	100	30	34	30	30	100	26	23	21	26	21	25	100	366
	100				124					142							

**Table 3:** Geographical distribution of haplotypes according to GenBank.

Haplotype	GenBank ID	State	N	Population status	N
H1	JQ350725	Korea	1	Native	4
	KR108208, MF594653, MF973559	China	3		
	KR482422, KR487647, KR489449, KR488759, KR488149, KR480162, KR480631, KR487306, HM411832, KT708724, KM849027, KM849820, KM850971, KM847661, MG054761, MG059719, MG060065, MG054902, MG061440	Canada	19	**Invasive**	71
	HQ978629-HQ978631, JF295977-JF296011	USA	38		
	KM448492, KM450721, KU907576, KU908539, KU908868, KU909840, KU910482, KU914359, KU915065, KU915323, KU915940, KU917742, KU918219, KU919113	Germany	14		
H2	MF594651, MF594652, MF973560	China	3	Native	3
H3	MF973574	China	1	Native	1
	KR486746, JF888326, MG299073	Canada	3	**Invasive**	3
H8	MF973566	China	1	Native	1
H9	MF973565	China	1	Native	1
H12	MF973562	China	1	Native	1
	KU917311	Germany	1	**Invasive**	1
H16	KC135950	Korea	1	Native	2
	MF973570	China	1		
H17	MF973568	China	1	Native	1
H22	MF973573	China	1	Native	1
H26-38	MF973561, MF973563, MF973564, MF973567, MF973569-MF973572, MF973575-MF973580	China	13 (1x13)	Native	13 (1x13)
H39	KU915107	Germany	1	**Invasive**	1
Total					104

Only 2 of the 39 known haplotypes–H1 and H3 –are found throughout all the range of *H*. *axyridis*. Haplotype H1 is the most massive. Its share is more than 70% in the entire sample analyzed. The total share of the H3 haplotype is also significant >7%. Haplotype H2 occurs with a frequency of 9.8%, but is distributed mainly in the populations of Siberia (the western group of native range populations). Only 3 samples of this haplotype were found in China and it was not found in any Russian populations of the eastern group of the native range. The frequency of the H9 haplotype does not exceed 2%, although it is widely distributed over the range and found in 6 populations analyzed by us (Table 2), as well as in China (Table 3). Haplotype H12 is even more rare, but found in the three populations analyzed in our study, as well as in China and Germany. The remaining 27 haplotypes are even less common and were registered in single individuals only.

Thus, the highest qualitative diversity of haplotypes is observed in the eastern group of native populations, where 30 haplotypes were found. Twenty-four three of them, including 17 unique ones, are found only in populations of this group. In the western group of native populations, only 11 haplotypes were revealed, 7 of which, including five unique ones, are found only in this group. The invasive group of populations has the lowest number of haplotypes. Only 6 haplotypes were found in it: two (H1 and H3)–common for the entire range, three haplotypes (H8, H9 and H12) were also found in native populations of the eastern group and one unique haplotype–H15. Another unique haplotype (H39) was found in Germany according to GenBank data (Table 2 and Table 3).

### Population genetic analysis

The sequences of *CO1* for *H*. *axyridis* from the GenBank database were excluded from population genetic analysis, since a significant part of these data has not yet been published, and it is impossible to establish the ratio of haplotypes in populations only according to GenBank.

We analyzed the variability of the *CO1* barcoding fragment in seven native populations of *H*. *axyridis* belonging to different geographical groups and in six invasive populations. The results of this analysis of the studied populations/population groups are shown in Table 4.

**Table 4:** Indices of variability of *H*. *axyridis* populations / population groups for polymorphism of the barcoding fragment.

Population status	Number of haplotypes	Molecular Diversity Indices			
		s*	π ± sd**	h (Hd) ± sd***	Pi ± sd****
**Native western population group**	**11**	**10**	**0.0026** ± 0.0017	**0.6485** ± 0.0293	**1.6867** ± 0.9967
Novosibirsk	5	5	0.0026 ± 0.0017	0.6873 ± 0.0475	1.6667 ±1.0024
Gorno-Altaisk	8	7	0.0027 ± 0.0018	0.6860 ± 0.0590	1.7290 ±1.0349
Sayan Mountains	4	5	0.0025 ± 0.0017	0.5625 ± 0.0495	1.6326 ± 0.9889
**Native eastern population group**	**14**	**12**	**0.0010** ± 0.0008	**0.5052** ± 0.0501	**0.6166** ± 0.4903
Birobidzhan	6	5	0.0008 ± 0.0008	0.4552 ± 0.1062	0.5057 ± 0.4413
Vladivostok	5	4	0.0005 ± 0.0006	0.3209 ± 0.1005	0.3422 ± 0.3468
The Russky Island	3	2	0.0008 ± 0.0008	0.5034 ± 0.0642	0.5264 ± 0.4526
The Troitsa Bay	7	5	0.0015 ± 0.0012	0.6759 ± 0.0770	0.9770 ± 0.6818
**Invasive**	**7**	**6**	**0.0003** ± 0.0004	**0.1612** ± 0.0418	**0.1666** ± 0.2260
Sochi	3	2	0.0002 ± 0.0004	0.1508 ± 0.0927	0.1538 ± 0.2219
Prague	4	3	0.0006 ± 0.0007	0.3834 ± 0.1196	0.4111 ± 0.3915
Berlin	3	2	0.0003 ± 0.0004	0.1857 ± 0.1102	0.1905 ± 0.2514
Kaliningrad	2	1	0.0001 ± 0.0003	0.0769 ± 0.0697	0.0769 ± 0.1532
Munich	1	0	0.0000 ± 0.0000	0.0000 ± 0.0000	0.0000 ± 0.0000
Denver	3	2	0.0002 ± 0.0004	0.1567 ± 0.0957	0.1600 ± 0.2270
**All populations**	**25**	**22**	**0.0016** ± 0.0011	**0.4907** ± 0.0303	**1.0320** ± 0.690

*”s” is the number of polymorphic sites (loci).

**“π” is the nucleotide diversity [53, 54]

***“h” (Hd) is the haplotype diversity [54].

****Pi is average number of pairwise differences between haplotypes [55].

To determine the hierarchical genetic structure in the groups of populations studied, specific fixation indices (Fst) [56, 57] were evaluated using molecular data for different levels of population grouping. The corresponding results are given in Table 5. The fixation index (Fst) of the analyzed populations without preliminary separation of the groups was more than 31%. When three groups of populations are distinguished, the Fst value increases even more, and when two groups are distinguished, they become the highest, reaching almost 50%. A specific index of population fixation in groups showed the absence of intragroup differentiation both in the group of invasive populations and in the western group of the native range. Fst in the group of eastern populations shows the presence of weak (about 7%), but significant (P-value = 0.00000 ± 0.00000) differentiation.”

**Table 5:** Fixation indices (Fst) for different levels of population association.

Population status	Populations	Specific population group Fst		
**Native western population group**	Novosibirsk	**0.01051** (P-value = 0.26588 ± 0.01512)	**0.01051** (P-value = 0.26588 ± 0.01512)	**0.31850** (P-value = 0.00000±0.00000)
	Gorno-Altaisk			
	Sayan Mountains			
**Native eastern population group**	Birobidjan	**0.07773** (P-value = 0.00000 ± 0.00000)	**0.07628** (P-value = 0.00000±0.00000)	
	Vladivostok			
	The Russky Island			
	The Troitsa Bay			
**Invasive**	Sochi	**0.01039** (P-value = 0.16129 ± 0.01046)		
	Prague			
	Berlin			
	Kaliningrad			
	Munich			
	Denver			
**Population group Fst**		**0.38060** (P-value = 0.00000±0.00000**)**	**0.49781** (P-value = 0.00000±0.00000)	

**Fig 3:**
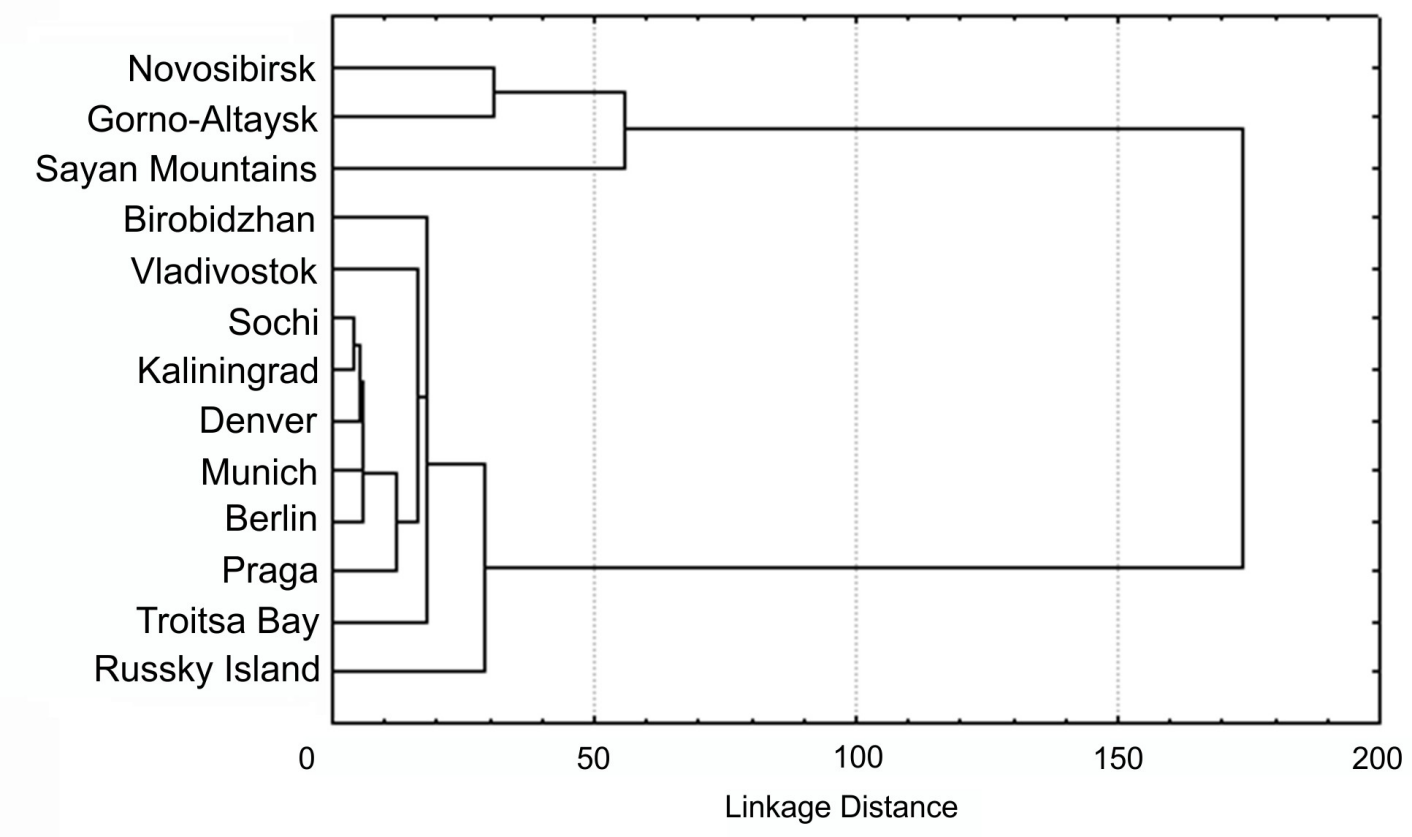
*H*. *axyridis* population cladogram, based on *CO1* gene haplotype frequencies. Linkage distance measured as Squared Euclidean distances.

To clarify and visualize relatedness between populations, we also performed a cluster analysis of samples by the distribution of haplotype frequencies shown in Table 3. The results of the cluster analysis are presented in Fig 3. The results of the cluster analysis presented in Fig 3 show the presence of two clusters remote from each other, one of which includes all populations of the western group of the native range. The second cluster contains all other populations, including invasive ones, the distances between which are minimal.

## Discussion

Our study of barcoding mtDNA fragment polymorphism in *H*. *axyridis* at the moment is perhaps the most representative for this species, both in the geographical spread of the populations and in the sample sizes from each of them. The nucleotide composition revealed in *H*. *axyridis* corresponds to the average for this fragment in coccinellids [58].

The total diversity of haplotypes that we found (25 among 366 individuals from 13 populations, 39 according to the Genbank data) is small in comparison with other species of ladybugs. For example, studies of the variability of the barcoding fragment in North American native ladybug beetles revealed 29 haplotypes among 149 *Hippodamia convergens* sequences, 9 haplotypes among 71 *Hippodamia parenthesis* samples and 4 haplotypes among 18 *Coleomegilla maculata* individuals [43]. The maximum intraspecific diversity of the barcoding fragment, as far as we know, was found in the recently described from the Iberian Peninsula highly specialized species of ladybirds *Iberorhyzobius rondensis*– 33 haplotypes among 104 samples [59]. Values of intraspecific indices of molecular variation, especially nucleotide diversity (π) (Table 4, bottom row) are also relatively small. These results may indicate a relatively recent (compared with other coccinellid species) separation of *H*. *axyridis* as a distinct species. This conclusion is concordant with the position of *H*. *axyridis* on the common phylogenetic tree of coccinellidae, constructed according to the results of multilocus typing [60].

### Haplotype phylogeny

The results of phylogenetic analysis of haplotypes carried out by different methods (Figs 1 and 2) are mostly congruent. The location of the H1, H2, and H3 haplotypes on dendrograms (Fig 1) and on the network (Fig 2) suggests that these haplotypes are the oldest for *H*. *axyridis*. The general geographical distribution of these haplotypes also confirms this assumption. The frequencies of H1 and H3 haplotypes in western native populations are significantly lower than in eastern ones. Haplotype H2, on the contrary, is the most abundant in western native populations, meeting here with a frequency of 43% (Table 2). This haplotype was also found in some native populations of the eastern group, namely, in two populations of eastern China (Table 3) in the provinces of Zhejiang [58] and Jiangsu (GenBank ID: MF973560) adjacent to the Coast seas. In the Russian eastern populations studied by us, this haplotype was not detected. Thus, the H2 haplotype is recorded in populations located in opposite parts of the species range and is absent, or it is very rare in populations located between them, which indicates their fixation in populations even before long-term isolation between eastern and western populations during the Sartan glaciation (25–10 thousand years ago) [13, 14, 18–20]. The same can be said about the rare H22 haplotype found in Novosibirsk (western group of populations, Table 3) and in China (GenBank ID: MF973573).

Thus, it is obvious that the haplotypes H1, H2, H3, and H22 appeared and were fixed in *H*. *axyridis* in sufficient quantities even before the beginning of the isolation processes of the Pleistocene-Holocene period. No less ancient, obviously, is the H4 haplotype, phylogenetically associated with H1 and H2. Its absence in the eastern group of native populations may be due to stochastic reasons.

### Population structure

Our results of assessing specific fixation indices (Fst) without preliminary identification of groups (Table 5) indicate the presence of a clear intraspecific subdivision of *H*. *axyridis* [56, 57]. The maximum value of the fixation index (Fst) for population groups is achieved with two groups: western native populations and a combined group including eastern native and all invasive populations. The results of cluster analysis of haplotype frequency distribution (Table 3), represented in Fig 3, show a similar population grouping. This indicates a high isolation of western native population group and confirms the hypothesis of all invasive populations originating from native populations of the eastern group.

A comparative analysis of molecular variability indices highlights various evolutionary scenarios population formation and their intraspecific groups [61]. The relatively high values of the molecular diversity of π and Pi in western native population group and lower in the eastern group (Table 4) with similar indicators of haplotype diversity (h) completely confirms the hypothesis of the microevolutionary history of the species, based on the analysis of complex morphological character geographical variation of *H*. *axyridis* in the [3, 13, 14]. In accordance with this hypothesis, modern native populations of the western group occurred in the Holocene from several small isolates that persisted in the regions of Western and Central Siberia during the peak of the last Pleistocene (Sartan) glaciation, and the eastern group of Russian populations occurred in the Holocene as a result of the expansion of the range of a fairly large eastern refugium formed during this glaciation in the territories corresponding to the modern southeastern provinces of China. The relatively high molecular diversity of western native populations can also be explained by the fact that a significant part of the haplotypes specific for this group comes from the H2 haplotype, which differs from the most common haplotype by three nucleotide substitutions for the H1 species (Fig 2, S1 Table). Russian populations of the group, descended from populations located on the border of species range expansion in the Holocene, apparently lost this haplotype, which became major in the native populations of the western group. The relatively high and similar values of haplotype diversity (h) in the western and eastern groups of native populations can be explained by different reasons: in western populations due to the origin from several small populations, and in eastern populations due to the appearance and accumulation of different single substitutions in different populations and gaps between them at the beginning of the process of range expansion due to isolation barriers.

Differences in specific fixation indices (Fst) of native populations within the western and eastern groups (Table 5) can probably be determined by their different population ages. Indeed, in the Far Eastern region, where the influence of cold was smoothed out by the coastal climate, warming after the last Pleistocene glaciation and, accordingly, the expansion of the H. axyridis range to the north, obviously, began much earlier than in the continental regions of Western and Central Siberia. The eastern populations, apparently, had enough time to accumulate and fix a reliable level of molecular differences. Evolutionarily younger populations of the western group, have not yet managed to accumulate the same level of molecular divergence. At the same time, the results of cluster analysis by haplotype frequencies (without taking into account their molecular diversity) (Fig 3) showed that the distances between western native populations are higher than between eastern ones. This result can be explained by the physical and geographical features of the habitat regions of these two groups of populations. Western populations live in high mountain regions, and migration between them is obstructed, at the same time there are no serious geographical barriers between eastern native populations. Differences in the frequencies of haplotypes between native populations of the eastern group, respectively, are mitigated due to effects of migration.

The group of invasive populations is the least diverse and differentiated according to all the parameters we studied. Only 6 haplotypes were revealed in it: two (H1 and H3)–common to the entire range, three haplotypes (H8, H9 and H12) were also found in native populations of the eastern group and one unique haplotype–H15. Another unique haplotype (H39) was found in Germany according to GenBank (Tables 2 and 3). Unique haplotypes detected in invasive populations are most likely to be found in native populations as well, as further studies are unlikely to have arisen and recorded over a relatively short invasion period of *H*. *axyridis*. All molecular diversity indices in the populations of this group are also significantly reduced, especially compared to native populations of the western group (Table 4). The value of the Fst index in this group (Table 5) does not show significant differences between its populations. The results of cluster analysis of samples only by haplotype frequencies (Fig 3) also indicate very low distances between invasive populations. Previously obtained data on the variability of the barcoding fragment in the American populations of *H*. *axyridis* [44] showed even lower levels of intra- and interpopulation variability.

Such a decrease in molecular variability in invasive populations and their high similarity among them is not a characteristic of all coccinellids introduced for biocontrol. For example, the level of molecular diversity of the invasive populations of seven-spotted ladybugs, which were actively released into nature in the United States and European countries, is quite high, comparable to native populations and correlated with the intensity of releases [62]. The low level of polymorphism of the barcoding fragment in invasive populations of *H*. *axyridis*, which was used no less actively than the seven-spotted ladybird for biocontrol, certainly confirms the random nature of its primary introduction [27], and the low level of interpopulation variability supports the origin hypothesis all invasive populations of *H*. *axyridis* from North American bridgehead populations [17, 45, 46]. However, our results do not support the hypothesis of the mixed origin of the eastern bridgehead, based on the analysis of the variability of microsatellites [17].

It should be noted that the conditional probability of the chosen scenarios for American bridgehead populations is very small– 0.4425 and 0.6242, respectively. At the same time, with the exception of two mitotypes (H1 and H3) common to populations of all groups, not one of the mitotypes found in the western group of native populations was found in invasive populations. Data on the phenotype of invasive populations also show their high similarity with native populations of the eastern group [22, 29, 41]. In addition, Lombaert et al. [17] used a sample from Almaty (Kazakhstan) as a potential source of invasion, considering this population as a representative of the western native range group. However, the origin of this population has not yet been clarified. It is removed from the documented boundaries of the native range of *H*. *axyridis* [3, 12, 34] as it could be formed due to the gradual penetration of small groups or several individuals of *H*. *axyridis* from Altai by rail and their hybridization with remnants of 250 thousand animals from Primorsky Krai (eastern group of populations) that were released in the Zailiysky Alatau at the end of the last century [34].

## Conclusions

Obviously, for a final answer to the questions of the native source (or sources) of bridgehead populations (ENA and WNA), further studies of the variability of various molecular and phenotypic markers in both invasive and native populations of *H*. *axyridis* are necessary. Of particular interest are the populations of China and Japan, for which data are currently not available in mass in scientific literature. However, the most intriguing question may be the fate of native populations of the Western group in the event of closure of the invasive range formed by natives of eastern populations. This question is not unfounded, since *H*. *axyridis* has already traveled east over 2,500 thousand kilometers from the place of the first find in Germany in 2001, and about 2100 km remained to go to the western border of the native range (the middle course of the Irtysh River in Western Siberia).

## Supporting information

S1 Fig Morphological features used earlier to analyze the intraspecific structure of Harmonia axyridis. The elytral ridge is a lateral chitin elevation at the distal ends of the elytra, inherited as a genetically determined monogenic trait. Absence of the ridge is determined by the recessive allele [63]. The elytral pattern is also inherited monogenically; it is controlled by the multi-allele locus with four main alleles and many minor alleles (total frequency <1% of the total) [64]. The population variability by this trait is traditionally evaluated by the ratio of the main four phenotypes. The pronotum pattern is a polymorphic trait, the inheritance of which is not yet understood. Variations of this trait in H. axyridis and the possibility of using it for inter-populational comparisons were earlier analyzed in detail by A.B. Blekhman [65].
(TIF)

S2 Fig Geographic variability of H. axyridis by elitral pattern and elitral ridge. (TIF)

S1 Table Localization and nature of nucleotide substitutions in barcoding mtDNA fragment (*CO1*) in *H*. *axyridis*. (DOCX)
