# Supplementary figures and images for: Variability of the mitochondrial CO1 gene in native and invasive populations of Harmonia axyridis Pall. comparative analysis
Source: PLoS One. 2020 Apr 2;15(4):e0231009. doi: 10.1371/journal.pone.0231009 (PMC7117877; doi:10.1371/journal.pone.0231009)

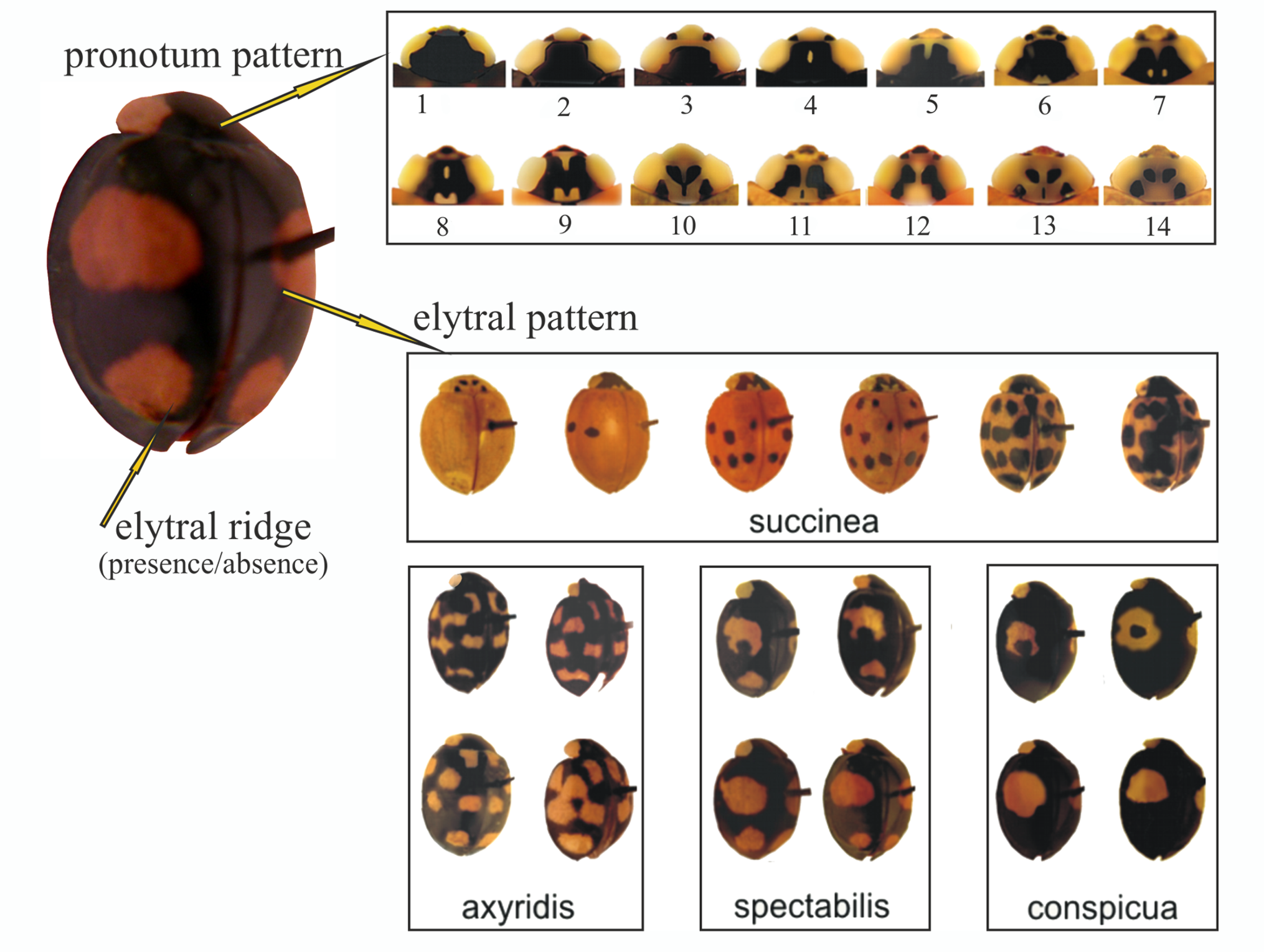

Supplement: S1 Fig — The elytral ridge is a lateral chitin elevation at the distal ends of the elytra, inherited as a genetically determined monogenic trait. Absence of the ridge is determined by the recessive allele [63]. The elytral pattern is also inherited monogenically; it is controlled by the multi-allele locus with four main alleles and many minor alleles (total frequency <1% of the total) [64]. The population variability by this trait is traditionally evaluated by the ratio of the main four phenotypes. The pronotum pattern is a polymorphic trait, the inheritance of which is not yet understood. Variations of this trait in H. axyridis and the possibility of using it for inter-populational comparisons were earlier analyzed in detail by A.B. Blekhman [65]. (TIF) [file pone.0231009.s001.tif]

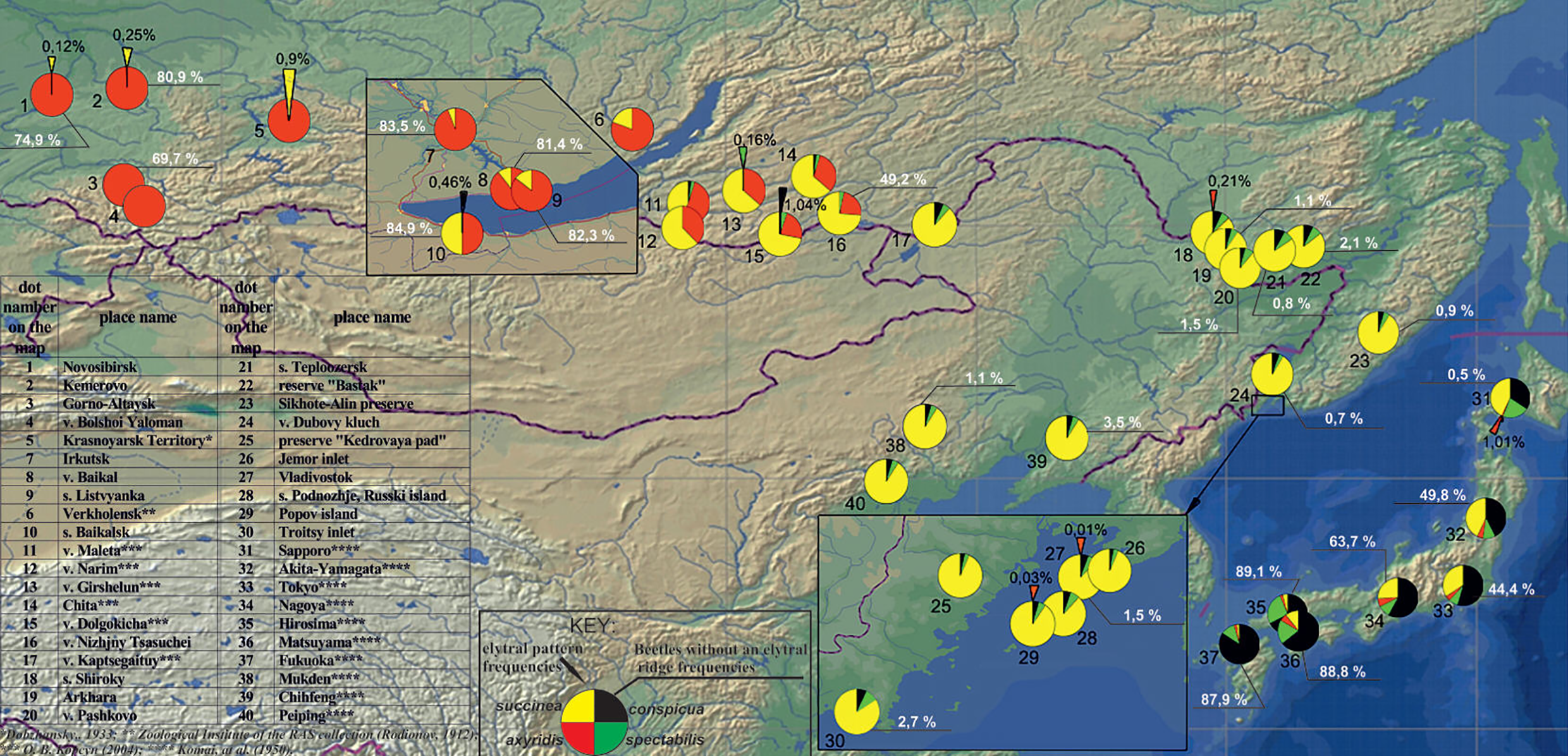

Supplement: S2 Fig — (TIF) [file pone.0231009.s002.tif]
