# Supplementary material for: Variability of the mitochondrial CO1 gene in native and invasive populations of Harmonia axyridis Pall. comparative analysis
Source: PLoS One. 2020 Apr 2;15(4):e0231009. doi: 10.1371/journal.pone.0231009 (PMC7117877; doi:10.1371/journal.pone.0231009)
Supplement: S1 Table — (DOCX) [file pone.0231009.s003.docx]

**S3 Table.** **Localization and nature of nucleotide substitutions in barcoding mtDNA fragment (*CO1*) in *H*. *axyridis*.**

| **Haplotype** | **GenBank ID** | **Polymorphic sites** | | | | | | | | | | | | | | | | | | | | | | | | | | | | | | | | | | |
| --- | --- | --- | --- | --- | --- | --- | --- | --- | --- | --- | --- | --- | --- | --- | --- | --- | --- | --- | --- | --- | --- | --- | --- | --- | --- | --- | --- | --- | --- | --- | --- | --- | --- | --- | --- | --- |
|  |  | 1307 | 1328 | 1337 | 1469 | 1478 | 1484 | 1538 | 1541 | 1565 | 1583 | 1592 | 1604 | 1613 | 1623 | 1628 | 1642 | 1652 | 1654 | 1661 | 1679 | 1701 | 1721 | 1725 | 1757 | 1784 | 1814 | 1817 | 1823 | 1838 | 1853 | 1865 | 1904 | 1907 | 1910 | 1916 |
| Reference sequence | KR108208 | **T** | **A** | **G** | **G** | **A** | **A** | **C** | **A** | **T** | **A** | **C** | **G** | **A** | **G** | **C** | **C** | **A** | **A** | **G** | **G** | **G** | **T** | **G** | **T** | **T** | **A** | **A** | **T** | **A** | **A** | **A** | **C** | **A** | **C** | **G** |
| **H1** | MN509563 | **T** | **A** | **G** | **G** | **A** | **A** | **C** | **A** | **T** | **A** | **C** | **G** | **A** | **G** | **C** | **C** | **A** | **A** | **G** | **G** | **G** | **T** | **G** | **T** | **T** | **A** | **A** | **T** | **A** | **A** | **A** | **C** | **A** | **C** | **G** |
| **H2** | MN509564 |  |  |  |  |  |  |  |  |  |  | **T** |  |  |  |  |  |  |  |  |  |  |  |  |  |  |  |  | **A** |  |  |  | **T** |  |  |  |
| **H3** | MN509565 |  |  |  |  |  |  |  |  |  |  |  |  |  |  |  |  |  |  |  |  |  |  |  |  |  |  |  |  |  |  |  | **T** |  |  |  |
| **H4** | [AM407723](https://www.ncbi.nlm.nih.gov/nucleotide/AM407723?report=genbank&log$=nuclalign&blast_rank=1&RID=37WY3271015) |  |  |  |  |  |  |  |  |  |  | **T** |  |  |  |  |  |  |  |  |  |  |  |  |  |  |  |  | **A** |  |  |  |  |  |  |  |
| **H5** | AM403713 |  |  |  |  |  |  |  |  |  |  | **T** |  |  |  |  |  |  |  |  |  | **C** |  |  |  |  |  |  | **A** |  |  |  | **T** |  |  |  |
| **H6** | MN509566 |  |  |  |  |  |  | **T** |  |  |  | **T** |  |  |  |  |  |  |  |  |  |  |  |  |  |  |  |  | **A** |  |  |  | **T** |  |  |  |
| **H7** | AM403712 |  |  |  |  |  |  |  | **G** |  |  | **T** |  |  |  |  |  |  |  |  |  |  |  |  |  |  |  |  | **A** |  |  |  | **T** |  |  |  |
| **H8** | AM407722 |  |  |  |  |  | **G** |  |  |  |  |  |  |  |  |  |  |  |  |  |  |  |  |  |  |  |  |  |  |  |  |  |  |  |  |  |
| **H9** | MN509567 |  |  |  |  |  |  |  |  |  |  |  |  |  |  |  |  |  |  |  |  |  |  |  |  |  |  | **T** |  |  |  |  |  |  |  |  |
| **H10** | MN509583 |  |  |  |  |  |  |  |  |  |  |  |  |  |  |  |  |  |  |  |  |  | **C** |  |  |  |  |  |  |  |  |  |  |  |  |  |
| **H11** | MN509568 |  |  |  | **A** |  |  |  |  |  |  | **T** |  |  |  |  |  |  |  |  |  |  |  |  |  |  |  |  | **A** |  |  |  | **T** |  |  |  |
| **H12** | MN509569 |  |  |  |  |  |  |  |  |  |  |  |  | **T** |  |  |  |  |  |  |  |  |  |  |  |  |  |  |  |  |  |  |  |  |  |  |
| **H13** | MN509570 |  |  |  |  |  |  |  |  |  |  |  |  | **T** |  |  |  | **T** |  |  |  |  |  |  |  |  |  |  |  |  |  |  |  |  |  |  |
| **H14** | MN509571 |  |  |  |  |  |  |  |  |  |  |  |  |  |  |  | **T** |  |  |  |  |  |  |  |  |  |  |  |  |  |  |  |  |  |  |  |
| **H15** | MN509572 | **G** |  |  |  |  |  |  |  |  |  |  |  |  |  |  |  |  |  |  |  |  |  |  |  |  |  |  |  |  |  |  |  |  |  |  |
| **H16** | MN509573 |  |  |  |  |  |  |  |  |  |  |  |  |  |  |  |  |  |  |  |  |  |  |  | **C** |  |  |  |  |  |  |  |  |  |  |  |
| **H17** | MN509574 |  |  |  |  |  |  |  |  |  |  |  |  |  |  |  |  |  |  | **A** |  |  |  |  |  |  |  |  |  |  |  |  |  |  |  |  |
| **H18** | MN509575 |  |  |  |  |  |  |  |  |  |  | **A** |  |  |  |  |  |  |  |  |  |  |  |  |  |  |  |  |  |  |  |  |  |  |  |  |
| **H19** | MN509576 |  |  |  |  |  |  |  |  |  |  |  |  |  |  |  |  |  |  |  |  |  |  |  |  |  |  |  |  |  |  |  |  |  | **T** |  |
| **H20** | MN509577 |  |  |  |  |  |  |  |  |  |  |  |  | T |  |  | **T** |  |  |  |  |  |  |  |  |  |  |  |  |  |  |  | **T** |  |  |  |
| **H21** | MN509578 |  |  |  |  |  |  |  |  |  | **T** |  |  |  |  |  |  |  |  |  |  |  |  |  |  |  |  |  |  |  |  |  |  |  |  |  |
| **H22** | MN509579 |  |  |  |  |  |  |  |  |  |  |  |  |  |  |  |  |  |  |  | **A** |  |  |  |  |  |  |  |  |  |  |  |  |  |  |  |
| **H23** | MN509580 |  |  |  |  |  |  |  |  |  |  | **T** |  |  |  |  |  |  |  |  |  |  |  |  |  |  |  |  | **A** |  |  |  | **T** |  |  | **A** |
| **H24** | MN509581 |  |  |  |  |  |  |  |  |  |  |  |  |  |  |  |  |  |  |  |  |  |  |  |  |  |  |  |  |  |  |  |  | **G** |  |  |
| **H25** | MN509582 |  |  |  |  |  |  |  |  |  |  |  |  |  |  |  |  |  | **C** |  |  |  |  |  |  |  |  |  |  |  |  |  |  |  |  |  |
| **H26** | MF973561 |  |  |  |  |  |  |  |  |  |  |  |  |  | **A** |  |  |  |  |  |  |  |  |  |  |  |  |  |  |  |  |  |  |  |  |  |
| **H27** | MF973563 |  |  |  |  |  |  |  |  |  |  | **T** |  |  |  |  |  |  |  |  |  |  |  | **A** |  |  |  |  | **A** |  |  |  | **T** |  |  |  |
| **H28** | MF973564 |  |  |  |  |  |  |  |  |  |  | **T** |  |  |  |  |  |  |  |  |  |  |  |  |  |  |  |  | **A** |  |  | **G** | **T** |  |  |  |
| **H29** | MF973567 |  |  |  |  |  |  |  |  |  |  |  | **A** |  |  |  |  |  |  |  |  |  |  |  |  |  |  |  |  |  |  |  |  |  |  |  |
| **H30** | MF973569 |  |  | **A** |  |  | **G** |  |  |  |  |  |  |  |  |  |  |  |  |  |  |  |  |  |  |  |  |  |  |  |  |  |  |  |  |  |
| **H31** | MF973571 |  |  |  |  |  |  |  |  | **C** |  | **T** |  |  |  |  |  |  |  |  |  |  |  |  |  |  |  |  | **A** |  |  |  | **T** |  |  |  |
| **H32** | MF973572 |  | **T** |  |  |  |  |  |  |  |  |  |  |  |  |  |  |  |  |  |  |  |  |  |  |  |  |  |  |  |  |  |  |  |  |  |
| **H33** | MF973575 |  |  |  |  |  |  |  |  |  |  |  |  |  |  |  |  |  |  |  |  |  |  |  |  | **C** |  |  |  |  |  |  |  |  |  |  |
| **H34** | MF973576 |  |  |  |  |  |  |  |  |  |  |  |  |  |  |  |  |  |  |  |  |  |  |  |  |  |  |  |  |  | **G** |  |  |  |  |  |
| **H35** | MF973577 |  |  |  | **A** |  |  |  |  |  |  |  |  |  |  |  |  |  |  |  |  |  |  |  |  |  |  |  |  |  |  |  |  |  |  |  |
| **H36** | MF973578 |  |  |  |  |  |  |  |  |  |  |  |  |  |  | **T** |  |  |  |  |  |  |  |  |  |  |  |  |  |  |  |  |  |  |  |  |
| **H37** | MF973579 |  |  |  |  |  |  |  |  |  |  |  |  |  |  |  |  |  |  |  |  |  |  |  |  |  |  |  |  | **T** |  |  |  |  |  |  |
| **H38** | MF973580 |  |  |  |  |  |  |  |  |  |  |  |  |  |  |  |  |  |  |  |  |  |  |  |  |  | **T** |  |  |  |  |  |  |  |  |  |
| **H39** | KU915107 |  |  |  |  | **G** |  |  |  |  |  |  |  |  |  |  |  |  |  |  |  |  |  |  |  |  |  |  |  |  |  |  |  |  |  |  |

* Non-synonymous substitutions leading to substitutions of amino acid residues in the protein sequence are highlighted with a gray background: at position 1307 phenylalanil is replaced by leucyl (H15); in 1583, leucyl by phenylalanil (H21); in 1592, isoleucil by methionyl (H18); in 1623 position – valil by isoleucil (H26); in 1642 position – seril by phenylalanil (H14, H20); in 1654 position – histidyl to prolil (H25); in the 1701 position – glycyl to arginine (H5) and in the 1725 position – valile to methionyl (H27).
